# Induction of Premature Labor

**Published:** 1904-11

**Authors:** Henry C. Coe

**Affiliations:** New York City


### INDUCTION OF PREMATURE LABOR.

BY HENRY C. COE, M.D., NEW YORK CITY.

It is the custom to apologize for introducing a hackneyed subject for discussion before a medical society, but on the present occasion I shall waive the usual introduction and merely state that I have intentionally chosen a familiar theme in order to emphasize the fact that in our anxiety to improve upon existing methods of procedure we are apt to undervalue those which have stood the test of experience.

While I am a firm believer in what my friend, Dr. Grandin, has aptly termed "elective" obstetrics, as opposed to the blind confidence of the older school in the unaided natural forces, I can not accept without protest the growing tendency to extend the indications for operative midwifery, so that it may keep pace with the development of modern surgery. In a well-equipped hospital operations are often deliberately elected and are successfully performed which would not be advised or permitted in private practice, where the circumstances, as regards both the condition of the patient and the aseptic environment, are essentially different. Cæsarian section, for example, which was once regarded as a formidable procedure, is now, under favorable aseptic conditions, rightly considered as attended with no more risks in skilled hands than an ordinary ovariotomy or abdominal hysterectomy. But our very success has led to an extension of the indications far beyond the limits originally prescribed for it. That we have been more conservative in New York than elsewhere is shown by the surprisingly small number of cases annually reported by the obstetricians as compared with those in neighboring cities possessing less clinical material. The same criticism applies, though in a lesser degree, to the relative frequency of the more common obstetric operations, such as version and high forceps.

We must infer either that natural delivery is each year becoming a more difficult process, whether by reason of actual dystocia or weakening of the ordinary expulsive forces, or that complications occur which might have been anticipated and prevented. Whatever explanation we may offer, it is clear that the field of obstetric surgery has been greatly extended during the past decade, as shown by the attention which is bestowed upon it in all the recent text-books. This is a subject of deep and never-failing interest to the practitioner. Though he may have no connection with the hospital work, and may be out of touch (except through his reading) with the advances in modern surgery, he can not be indifferent to the fact that midwifery is no longer an art but a science as well. Luck is a word no longer used in aseptic surgery. Why should it still govern the practice of midwifery?

The care of the pregnant female includes more than the perfunctory treatment of her minor ailments and an occasional superficial examination of her urine, no attention being paid to her pelvis, or to the size and position of the fetus until labor actually commences. It is beginning to be generally realized that the responsibility for fatal cases of eclampsia, for the death of the child during delivery, or for subsequent invalidism of the mother, like puerperal septicemia, can not be placed on fate or providence. The surgeon alone must assume the responsibility of his operation, the accoucheur of his case of obstetrics. This is perfectly just.

This leads us directly to the subject in hand, for only by the careful observation of our patient during the latter months of pregnancy can we recognize the danger-signals, anticipate serious complications, and thus guide to a fortunate conclusion a case which might otherwise terminate disastrously.

In introducing the question of premature labor, I desire to limit the discussion to those cases in which natural labor is anticipated by a month or less, primarily in the interests of the mother, but also with the view of saving the fetus at a time when there can be no question as to its viability. This includes cases of rapid delivery, or *accouchment force,* which has its own proper indications. In order to meet the reproach that this practice might easily be extended beyond the somewhat ill-defined limits which divide elective obstetrics from "meddlesome midwifery," I would state at the outset that such interference with pregnancy is not to be lightly undertaken, nor is it entirely free from risk. It is an operation, and is to be conducted like one. It may not be amiss here to emphasize the fact that the accoucheur is not expected to consult his own interest, or the mere convenience of the patient with regard to the proper time at which labor be induced. This is rather too advanced even for this rapid age, although it has been advocated in this city.

Between this rash and unjustifiable interference without any reasonable indications and the unaccountable timidity of many practitioners, who hesitate and temporize until too late to effect the desired result, there is a middle course which is the proper one for the scientific obstetrician.

I do not intend to discuss all the obvious indications for this operation which are laid down in the text-books, such as placenta previa, accidental hemorrhage, advanced cardiac and pulmonary disease, serious nervous affections, etc., but to confine myself to cases in which the mother is in good health up to the latter weeks of pregnancy, and the fetus is alive. Habitual death of the latter in the last month would naturally furnish a clear indication, provided of course that anti-syphilitic treatment had been pushed without success. The two indications which I regard as of most practical importance, and which are commonly overlooked, are renal insufficiency and a disproportion between the head and the parturient canal.

So much has been said about the importance of routine urinary analysis, especially during the last weeks of pregnancy, that I feel that it is unnecessary to enter into details. If there is any one clinical fact which has been impressed upon my mind during the past year it is that uremic symptoms (like the discharges in cancer of the uterus) unusually represent not an initial process, but a culmination. Albuminuria, and even marked diminution of the daily amount of urine, are like the torpedoes which it is hoped may arrest the train after the danger-signal has been passed unheeded. Progressive diminution in the amount of urea (with or without the appearance of casts) in spite of careful regulation of the diet, diuretics, purgatives, etc., it is a clear indication that the patient is not going to improve as the pregnancy continues. I have learned from sad experience that if we wait for evidences of general toxemia, for headache, edema, dyspnea and the familiar train of symptoms, we *may* wait too long. This was brought home to me most forcibly by an apparently simple case in which, without warning, the patient had an eclamptic seizure while I was dilating the cervix preparatory to inserting gauze, and died in spite of heroic treatment.

It is in the case of primiparæ that we are most likely to be caught napping, since we would naturally be on the lookout for trouble in a multipara with a history of former renal complications. Not to weary you with clinical histories, I shall simply state that within a year I have had six cases in which labor was successfully induced on this indication—persistent and progressive diminution of urea during the last month. An occasional trace of albumin and a few casts appeared in two or three, and the amount of urine was seldom diminished below 600 or 800 cc., but the evident toxemic condition was the one which in my opinion justified interference. Nor must we forget that the child is in jeopardy as well as the mother. Although no evidence may be afforded that its vitality is impaired, it is natural to infer that the vitiated blood of the mother acts as a veritable poison to the growing fetus, so that it is less able to survive a normal, still more, an artificial delivery. We are thus more likely to sacrifice the child by delay, while the mother's chances are not improved.

It goes without saying that in private practice patients are usually averse to interference when they feel perfectly well, and fear that the premature child will die in consequence. Nervous women and their families may be considerably disturbed at the recommendation, but I have never found that their objections were insuperable, especially when other counsel was obtained.

The other common indication, disproportion between the fetus and pelvis, is not so clear. Its recognition implies frequent examinations after the seventh month, careful pelvic measurements, and attempts to estimate the relative size of the head and its adaptation to the birth-canal. I do not refer in this connection to obvious pelvic contraction, but to those minor degrees of antero-posterior flattening, marked false promontory, etc., which we usually encounter in private practice, and which would possess no importance if the child were of small size. But if the fetus, even at the eighth month, is plainly above the average size, and the mother is suffering from severe pressure symptoms, the propriety of interference a couple of weeks before term may be reasonably entertained. In the case of a multipara, with a history of previous difficult labors, our course is clear, but when we have to do with a primipara we must often be in doubt as to the proper action. To lose the child under these conditions would be awkward for the practitioner who urged the induction of premature labor, but who of us after a difficult instrumental delivery, resulting in the death of the child and the extensive laceration of the maternal tissues, has not wished that he had anticipated nature? In fact, intelligent women not infrequently ask why this was not done, feeling, as they naturally should, that the modern accoucheur ought to be able to foresee and prevent such complications. In spite of the difficulty of accurately estimating the size of the fetal head in utero and even of certainly recognizing abnormal positions, I believe that both accoucheur and patient will regard the introduction of labor two or three weeks before term as a rational procedure in cases such as I have described. That the operation may easily be abused, I admit. It presupposes a careful and conscientious study of the conditions and a clear understanding on the part of the mother, that the interests of the child are considered as well as her own.

Many other points will occur to you in connection with the two indications which I have mentioned, but I leave these to be brought out in the discussion. As regards prognosis, we are justified in giving an entirely favorable one for the mother, so far as the risks of the operation and subsequent labor are concerned. To guard ourselves, it is better not to promise too confidently that the child will survive, especially in uremic (or more properly toxemic) cases. It must be impressed upon the family that the fetus is premature, and that while it may not be necessary to keep it in an incubator, it will require constant care for several weeks, particularly in the matter of feeding.

I have nothing new to offer with regard to technique. I wish that I had, for there is room for improvement upon our existing methods. In cases in which we aim simply to anticipate, not to precipitate, delivery I assume that our object is to imitate Nature as closely as possible by causing softening and dilatation of the lower uterine segments, as well as intermittent contractions. To my mind the former is by far the more important, especially in view of the necessity of rapid emptying of the uterus which may arise. It would seem to be self-evident that in premature labor violent uterine contractions with a rigid, undilated cervix, and perhaps escape of the amniotic fluid, are most undesirable results of our interference. It is, therefore, a matter of surprise to find high authorities advocating the time-honored practice of introducing a bougie into the uterus as the *only* method of inducing labor which they can recommend. I would be glad to learn the views of my hearers on this point. Personally, I have abandoned it, as it has been most unsatisfactory in my hands. Aside from the danger of premature rupture of the membranes, I have not found that contractions invariably follow, even when the bougie remains *in situs* from twenty-four to forty-eight hours. As I have shown in a former paper, the mere presence of a foreign body within the pregnant uterus, whether a bougie, a water-bag, gauze-tampon, or even the hand, does not by any means imply that the uterine muscle will promptly respond to the irritation. In my own practice the gauze-tampon has been most satisfactory, although I would insist that too little gauze is introduced as a rule. Instead of inserting five or six feet, as most of the books advise, I rarely use less than three or four yards, aiming to thoroughly distend the lower segment.

To linger over the details of this simple operation would be a waste of your time. I only wish to emphasize briefly certain points. Rigid asepsis, a good light, sufficient assistance and control of the patient are necessary to success, as in any other gynecological operation. We can not afford to slur over any details. An anesthetic may or may not be necessary, according to the disposition of the patient and the difficulty of exposing the os. Nitrous oxide or chloroform in skilled hands is employed. I have seldom found it necessary, though it must be admitted that the preliminary scrubbing of the external genitals and vagina is trying, even for the most stolid or courageous woman. The actual operation is the shortest step in the procedure. Let me protest against the free and easy method of inserting the gauze by the touch alone, with imperfect asepsis and uncertain location of the tampon. This introduces an element of chance into an otherwise harmless procedure, which must necessarily bring it into disrepute.

A careful examination (especially if the patient is anesthetized) is advisable before beginning the preliminary dilatation. That it is important to locate the placenta, if possible, and also to push the gauze along the posterior uterine wall, was illustrated by a case of accidental hemorrhage which I reported recently to the Obstetrical Society, where a low-placed placenta on the anterior wall was partly detached after labor began, probably by pressure of the gauze tampon. Again, too great force must be avoided when pushing in the gauze, in order to prevent rupture of the membranes. The objection to the rubber bags, that they displace the presenting part, can not be urged against the gauze if properly applied. I seldom expect any progress in less than twenty-four hours. The patient is allowed to be up and about. Quinine is pushed to the extent of ten grains every six or eight hours, according to the tolerance of the patient, with strychnin gr. 1-40. It is my routine practice to give strychnin during the last three months of pregnancy. If pains are slight and irregular, the gauze may be left thirty-six hours, but I usually remove it earlier, substituting a good-sized bag, which I have always found to be efficient and harmless, in spite of objections which have been raised to its use. Since I began to use quinine I have not had occasion to introduce a bag after removing the gauze, as after removal of the tampon the fetal head had descended into the dilated lower segment and labor was fairly in progress. I can see no advantage in adopting the advice given in a recent work on obstetrics that "the tampon must be removed daily until labor pains appear." (*Modern Obstetrics*, Dorland, p. 277.)

So far I have made no reference to manual dilatation. Should the physical condition or morale of the patient, or the interests of the fetus lead us to decide that it would be unwise to prolong the period of waiting, the opportunity for this procedure is most favorable, owing to the softening of the tissues in consequence of the continued pressure of the tampon. Under anesthesia, and with the same aseptic preparations as before, the cervix may be dilated by the method described by Edgar, and labor may then be allowed to proceed, or, if more rapid delivery is deemed advisable, after thorough dilatation and *paralysis* of the sphincter muscle, version can be performed. The latter course would certainly be preferable in uremic conditions. In my last case of this character, a few days ago, the increasing gravity of the symptoms at the time of removing the gauze did not justify an hour's delay, and as the cord had ceased to pulsate, when I introduced my hand I at once performed craniotomy and extraction of the small fetus with good results.

Other methods of inducing premature labor have been suggested, but they have not stood the test of experience. I have been tempted to try the mild faradic or interrupted galvanic current (not the constant, as recommended by Bayer) to assist the action of the tamponade, but have hesitated about employing this agent, because of the objection that the life of the fetus might be imperiled.

If the artificial induction of labor a few weeks before term is such a simple procedure and so uniformly successful, the question naturally suggests itself, "Why is it not more popular with the profession?" So far as my observation goes, this is due to several causes, the chief of which is timidity, over-caution, or ultra-conservatism on the part of the attendant, the same motives which are responsible for so many fatal cases of ectopic pregnancy and appendicitis—the fear of being blamed if anything goes wrong. This has caused many a man to temporize when his own common sense warned him that such delay was fatal. Lastly (and this can not be excused as an error of judgment), the real reason why elective obstetrics is still in its infancy is that we do not yet justly appreciate the fact that our responsibility to the pregnant female begins long before parturition. This is indeed the essential difference between the rôle of the scientific physician and that of the midwife.—*International Journal of Surgery.*

